# Whole-Genome Resequencing-Based GWAS Reveals Major-Effect Loci and Candidate Genes for Growth Traits in Topmouth Culter (*Culter alburnus*)

**DOI:** 10.3390/ani16131969

**Published:** 2026-06-25

**Authors:** Wenping Jiang, Junzhi Luo, Jianbo Zheng, Shili Liu, Meili Chi, Shun Cheng, Chao Zhu, Xiaoying Hang, Miao Peng, Fei Li

**Affiliations:** Key Laboratory of Genetics and Breeding, Zhejiang Institute of Freshwater Fisheries, Huzhou 313001, China; jordanping23@icloud.com (W.J.); luojunzhi3216@163.com (J.L.); 21207054@zju.edu.cn (J.Z.); liushili1212@126.com (S.L.); chimeili83404109@126.com (M.C.); sschengshun@sina.com (S.C.); chaozhu5739@163.com (C.Z.); runtogalaxy8848@gmail.com (X.H.); pengmiao614@163.com (M.P.)

**Keywords:** *Culter alburnus*, growth-related traits, genome-wide association study, whole-genome resequencing, SNP

## Abstract

Topmouth culter (*Culter alburnus*) is one of the most economically important freshwater fish in China, but intensive aquaculture has caused germplasm degradation and reduced growth performance, while the genetic basis underlying growth variation in this species remains poorly understood. This study aimed to identify major-effect loci and candidate genes associated with growth-related traits to support molecular breeding. Whole-genome resequencing (average depth 11.44×) was performed on 300 individuals derived from random mating among three geographic populations (Danjiangkou, Taihu, and Poyang Lake); 239 individuals with complete phenotypic records were retained for a genome-wide association study (GWAS) of five growth-related traits, including body weight (BW), body weight without viscera (BWW), total length (TL), body length (BL), and body height (BH). After stringent filtering, 7,597,008 high-quality single-nucleotide polymorphisms (SNPs) were obtained, and association analysis was conducted using a linear mixed model, followed by Benjamini–Hochberg false discovery rate correction and 1000-permutation testing for BW and BL. Six genome-wide significant SNPs and 473 suggestive SNPs were identified, with individual significant SNPs explaining over 11% of phenotypic variance, indicating candidate loci of putatively moderate-to-large effect. Significant SNPs were predominantly clustered on chromosomes 16 and 19. Four candidate genes—*aig1*, *cacna1b*, *pgm5*, and *bcr*—were identified, with functions related to lipid metabolism, muscle structure, and cell proliferation. This first population-level GWAS in topmouth culter provides valuable molecular markers for marker-assisted selection and lays a foundation for accelerated genetic improvement of this species.

## Simple Summary

Topmouth culter is a popular freshwater fish in China, valued for its tender flesh and delicate flavor, and an important species for aquaculture. However, years of intensive farming have caused problems such as slower growth and smaller body size, which seriously affect fish farmers and the industry. Faster-growing fish would mean better production efficiency, lower costs for farmers, and a more stable food supply for consumers. To address this, we set out to find the inherited factors that influence how big these fish grow. We measured five growth-related traits—including body weight and body length—in 239 individual fish. By comparing differences among the fish with their growth performance, we identified six key inherited markers strongly linked to growth, along with four candidate factors that likely play important roles in regulating body size, fat use, and muscle development. These findings provide useful tools that breeders can use to select young fish with greater growth potential, helping to develop improved varieties, support sustainable aquaculture, and benefit both fish farmers and consumers.

## 1. Introduction

Topmouth culter (*Culter alburnus* Basilewsky, 1855; synonym *Erythroculter ilishaeformis*) belongs to the order Cypriniformes, family Xenocyprididae, and genus *Culter*. It is a large predatory freshwater fish widely distributed across major rivers, lakes, and reservoirs throughout China, and is also found in the Russian Far East, Mongolia, and the Korean Peninsula [1]. Renowned for its tender flesh, delicate flavor, and rich nutritional profile, topmouth culter is recognized as one of the “Four Famous Freshwater Fishes” of China and holds a prominent position in the Chinese freshwater aquaculture industry. However, with the intensification of aquaculture practices, the combined effects of limited broodstock populations, high-density rearing, and inbreeding have led to marked germplasm degradation in farmed stocks [2]. Its most prominent manifestation is a decline in growth performance, manifested at slower growth rates and progressive miniaturization of body size [2,3]. Counteracting this growth decline through genetic improvement, therefore, requires, as a first step, a clear understanding of the genetic basis of growth-related traits in topmouth culter.

Genome-wide association study (GWAS), as a powerful genomic tool, enables the systematic detection of associations between genome-wide genetic variants and phenotypic traits, thereby facilitating the identification of candidate genes and molecular markers affecting complex trait variation. It has become a core approach for dissecting the genetic basis of economically important traits in aquatic animals. Compared with traditional family-based linkage analysis (QTL mapping), GWAS offers broader genomic coverage and higher mapping resolution, making it particularly suitable for detecting common variants underlying quantitative trait variation in natural or breeding populations.

In recent years, GWAS has achieved notable progress in growth trait studies of aquaculture fish species. In the closely related cyprinid grass carp (*Ctenopharyngodon idella*), Hao et al. [4] identified five SNPs significantly associated with body weight, body length, and body height, while Zhang et al. [5] discovered 31 significant SNPs through whole-genome resequencing, involving candidate genes such as *pwp1*, *cers5*, and *chrebp*. In other aquaculture species, growth-associated SNPs have been reported in the largemouth bass (*Micropterus salmoides*) [6], giant grouper (*Epinephelus lanceolatus*) [7], Atlantic salmon (*Salmo salar*) [8], and Nile tilapia (*Oreochromis niloticus*) [9,10]. These studies collectively demonstrate that GWAS is a powerful approach for dissecting the genetic architecture of growth-related traits and accelerating molecular breeding in cultured fish species.

Translating such genomic insights into practical breeding gains is particularly valuable for growth-related traits, which are among the most economically important traits in aquaculture and the primary objective of most fish breeding programs [11,12,13]. These traits are especially well-suited to marker-assisted and genomic selection for several reasons. First, they are typical polygenic quantitative traits with substantial additive genetic variance and moderate-to-high heritability, so they respond efficiently to selection, and their genetic merit can be predicted by aggregating effects across many markers [11,13]. Second, several economically key growth-related traits, most notably body weight without viscera, a proxy for carcass/processing yield, cannot be recorded directly on the live breeding candidates because they require slaughter; marker- and genome-based information is therefore essential to select for them indirectly through information on relatives [14]. Third, because body size is expressed relatively late in the production cycle, genomic prediction enables selection candidates to be ranked earlier, shortening the generation interval [14]. Although conventional phenotypic selection can improve these traits to some extent, it is constrained by long breeding cycles, low selection efficiency, and difficulty in accurately assessing genetic potential; marker-assisted selection (MAS) and genomic selection (GS), which rely on exactly the kind of markers and loci that GWAS provides, have therefore been increasingly adopted in aquatic animals to enhance breeding efficiency and genetic gain [11].

Despite its importance to the Chinese freshwater aquaculture industry, the genetic basis of growth-related traits in topmouth culter remains relatively poorly understood. Previous studies have focused mainly on germplasm resource evaluation [2,15], chromosome-level genome assembly [3,16,17], and hybrid breeding [18]. Efforts to dissect growth-related traits genetically have so far relied on family-based linkage mapping and candidate-gene/marker-association approaches: only Liu et al. mapped 12 QTLs for body weight using family-based linkage maps [3]; Chi et al. identified three *smad4* SNPs associated with growth in a gynogenetic population through a candidate-gene approach [19]; Liu et al. characterized two growth hormone receptor (*ghr*) genes and detected microsatellite polymorphisms associated with growth-related traits [20]; and Cheng and Sun reported myostatin (*mstn*) polymorphisms associated with growth in a *Culter alburnus* × *Ancherythroculter nigrocauda* hybrid [21]. However, linkage and single-marker/candidate-gene analyses are inherently limited by restricted recombination and narrow genetic backgrounds in the former, and by the small number of loci interrogated in the latter, which constrains their power to capture genome-wide minor-effect variants and their mapping resolution. To date, no population-level, genome-wide association study has been reported for this species. With the recent chromosome-level genome assemblies of topmouth culter and the broad adoption of high-throughput sequencing, a population-level GWAS to comprehensively dissect the genetic architecture of growth-related traits has become a priority for identifying major functional loci and advancing MAS.

Accordingly, the present study aimed to analyze the genetic basis of body-size variation in topmouth culter and to identify major-effect loci and candidate genes that can underpin its molecular breeding. Because the experimental fish formed a single contemporaneous cohort reared communally to a uniform age of 18 months, body size at this age reflects cumulative growth and serves as the principal growth-related selection criterion in fish, in line with standard practice in aquaculture breeding [12]. On this basis, five growth-related traits were assessed, including body weight (BW), body weight without viscera (BWW), total length (TL), body length (BL), and body height (BH). To dissect their genetic architecture, we performed a population-level genome-wide association study (GWAS) on whole-genome resequencing data from 239 such individuals, generated by random mating among three geographic populations (Danjiangkou, Taihu, and Poyang Lake), and prioritized candidate genes from the associated loci, thereby providing a molecular foundation for marker-assisted and genomic selection in this economically important species.

## 2. Materials and Methods

### 2.1. Experimental Animals

The broodstock of topmouth culter used in this study were sourced from three geographically distinct wild populations in China: Danjiangkou Reservoir, Hubei Province (*n* = 45), Taihu Lake, Jiangsu Province (*n* = 40), and Poyang Lake, Jiangxi Province (*n* = 46), totaling 131 broodstock. The broodstock were collected during August 2020 and transported to the Deqing Comprehensive Experimental Base of the Zhejiang Institute of Freshwater Fisheries (Deqing County, Zhejiang Province, China). All broodstock were communally reared in outdoor earthen ponds (1334 m^2^, water depth 2.0 m) under natural photoperiod conditions. Water quality parameters were monitored regularly and maintained within the following ranges: culture water temperature 10–32 °C (seasonal variation: ~10 °C in winter to ~32 °C in summer), dissolved oxygen ≥ 5.0 mg/L, pH 7.0–8.0, ammonia nitrogen ≤ 0.2 mg/L, and nitrite ≤ 0.05 mg/L. Fish were fed a commercial formulated diet at a daily feeding rate of 4% body weight, administered twice daily (08:00 and 16:00). During the spawning season (June 2021), the pooled broodstock from the three populations were allowed to reproduce by communal natural (mass) spawning in a shared pond, and 300 of the resulting offspring were randomly selected as the experimental cohort for whole-genome resequencing and subsequent GWAS analysis. Because mating was not controlled and no parental genotyping or parentage assignment was performed, the reproductive contributions of individual broodstock—and, by extension, of the three source populations—were not quantified and are likely to have been unequal, as is typical of communal/mass-spawning systems in *C. alburnus* and other cultured fishes [22,23]. Accordingly, the offspring were analyzed not as a balanced three-population cross but as a single synthetic, admixed cohort, and the attendant variance in family size and cryptic relatedness was explicitly modeled in the association analysis through a genome-wide kinship matrix and the leading principal components (see Section 2.5, Section 2.6, Section 3.3 and Section 4.2). The offspring were reared communally in outdoor earthen ponds (1334 m^2^, water depth 2.0 m) at the same facility under the same water quality and feeding management conditions described above, with stocking density maintained at approximately 30,000 individuals per hectare.

### 2.2. DNA Extraction, Library Construction, and Sequencing

A total of 300 juvenile topmouth culter (3 months post-fertilization, 3 mpf) were anesthetized using MS-222 (ethyl 3-aminobenzoate methanesulfonate, 50 mg/L), after which approximately 20–30 mg of caudal fin tissue was excised and preserved in 100% anhydrous ethanol at 4 °C for genomic DNA extraction. The juveniles were subsequently tagged with passive integrated transponder (PIT) microchips and returned to the ponds for continued communal rearing. Each fin-tissue sample was individually labeled and linked on a one-to-one basis to the unique PIT-tag code implanted in the same fish. This PIT identity was used throughout the study to match each individual’s whole-genome resequencing data to the phenotypic records collected at 18 mpf, thereby ensuring full traceability between the DNA samples and the reared specimens. Genomic DNA was extracted using the DNeasy Blood & Tissue Kit (69504; Qiagen, Hilden, Germany) according to the manufacturer’s instructions. DNA quantity and quality were assessed using a NanoDrop One spectrophotometer (840-317400; Thermo Scientific, Waltham, MA, USA) to evaluate the A260/A280 ratio and A260/A230 ratio, Qubit 3.0 fluorometer (Q33218; Invitrogen, Carlsbad, CA, USA) for accurate quantification, and Agilent 2100 Bioanalyzer (Agilent Technologies, Santa Clara, CA, USA) to assess DNA integrity. Only DNA samples with a total amount ≥ 1.0 μg and no apparent degradation were used for library construction. Whole-genome sequencing libraries were constructed using the UltraClean Rapid DNA Library Prep Kit for MGI (UNDM811; Vazyme, Nanjing, China) with an insert size of approximately 350 bp. Briefly, qualified genomic DNA was fragmented by sonication, followed by end repair, A-tailing, adapter ligation, and PCR amplification (5 cycles). Library quality was evaluated using the Agilent DNA 1000 kit on the Agilent 2100 Bioanalyzer, and library concentration was quantified by quantitative real-time PCR (qPCR). Qualified libraries were pooled and sequenced on the MGI-2000/MGI-T7 platform (MGI, Shenzhen, China), generating paired-end reads of 150 bp (PE150).

### 2.3. Measurement of Growth-Related Traits

At 18 mpf, individual identity was confirmed by scanning the PIT microchip tags using a handheld reader, and then experimental fish were euthanized by immersion in an ice-water slurry. The following five growth-related traits were subsequently measured and recorded: body weight (BW, g), body weight without viscera (BWW, g), total length (TL, cm), body length (BL, cm), and body height (BH, cm). Body weight and body weight without viscera were measured using an electronic balance (LYP-10002E; LABGIC, Beijing, China), while length and height traits were measured using a digital vernier caliper (YHT kachi 53723433581; Yanhengtong, Shenzhen, China). All measurements were performed by the same trained personnel to minimize measurement error. Because each fish’s phenotype could be linked to its resequencing data only through its PIT-tag identity, only individuals that were successfully identified by PIT-tag scanning at 18 mpf could be included in the analysis. Of the 300 sequenced individuals, 239 (79.7%) were successfully identified, phenotyped, and retained for the GWAS, whereas the remaining 61 (20.3%) could not be detected by PIT-tag scanning at harvest—owing to tag loss or to non-recovery of individuals (including mortality) over the grow-out period—and were therefore excluded. A cumulative non-detection rate of this order over a multi-month grow-out is consistent with the substantial long-term PIT-tag loss documented in fish [24].

### 2.4. SNP Discovery and Genotyping

Raw sequencing data were subjected to stringent quality control using fastp (v0.20.0) [25] to obtain high-quality, clean reads. Filtering criteria included: (1) removal of reads containing sequencing adapters; (2) removal of reads with an N content exceeding 10%; and (3) removal of low-quality reads in which more than 40% of bases had Phred quality scores ≤ 20. Clean reads from each sample were aligned to the topmouth culter reference genome (CNCB, GWHBOSX00000000; genome size, 1.05 Gb) using BWA-MEM (v0.7.17) [26] with default parameters. Variant calling was performed using GATK (v4.0.4.0) [27]. Briefly, per-sample candidate variants were identified using the HaplotypeCaller tool in GVCF mode. The resulting GVCFs were then consolidated with CombineGVCFs and jointly genotyped across all 300 samples using GenotypeGVCFs. Hard filtering was applied to the raw SNP set using the following GATK criteria: QD < 2.0; QUAL < 30.0; FS > 60.0; MQ < 40.0; MQRankSum < −12.5; ReadPosRankSum < −8.0; SOR > 3.0, resulting in 25,975,940 SNPs. Subsequently, more stringent quality control was performed using PLINK (v1.90) [28] with the parameters: --geno 0.02 --mind 0.1 --maf 0.05 --hwe 1 × 10^−6^. After these filtering steps, all 300 samples were retained, and a final set of 7,597,008 high-quality SNPs was obtained for downstream analyses. The genomic location and predicted functional impact of these SNPs were subsequently annotated using ANNOVAR (v2019-10-24) [29] based on the reference genome annotation.

### 2.5. Analysis of Population Structure and Genetic Relatedness

Because cryptic relatedness and population stratification can confound association tests and inflate false-positive rates in GWAS [30], we first characterized the genetic structure of the experimental cohort so that it could be appropriately accounted for in the association model. Multidimensional population genetic analyses were performed based on the filtered high-quality SNPs. To reduce the confounding effects of linkage disequilibrium, SNPs were pruned using PLINK (v1.90) [28] with the parameters --indep-pairwise 50 5 0.2 prior to population structure analyses. Principal component analysis (PCA) was conducted using GCTA (v1.26.0) [31] to assess population stratification, and the first two principal components (PC1 and PC2) were plotted to visualize genetic clustering. A phylogenetic tree was constructed using the neighbor-joining method in MEGA (v12.1) [32], with bootstrap support evaluated over 1000 replicates. Population genetic structure was evaluated using ADMIXTURE (v1.3.0) [33], with K values set from 1 to 20 and 10 independent runs per K value. The optimal K was determined based on the minimum five-fold cross-validation error rate (CV error). A kinship matrix was generated using GEMMA (v0.98.3) [34] to correct for false positives in subsequent association analyses. Linkage disequilibrium (LD) analysis and *r^2^* calculations were performed using PopLDdecay (v3.41) [35], with a maximum distance of 300 kb.

### 2.6. Genome-Wide Association Study on Growth-Related Traits

GWAS for each of the five growth-related traits was performed independently using GEMMA (v0.98.3) [34] based on the following linear mixed model:

(1) y=Wα+xβ+u+ε

where *y* denotes the vector of phenotypic values; *W* is the covariate matrix including the intercept and the first 10 principal components (PCs); *α* represents the corresponding fixed-effect coefficients; *x* is the genotype vector of the tested SNP; *β* is its effect size; *u* is the vector of random polygenic effects following *u* ~ *MVN*(0, *λτK*), where *K* is the centered relatedness matrix; and *ε* is the residual error vector. The fixed effects in W comprised the overall intercept and the first ten genome-wide principal components (PCs), the latter included to correct for population stratification and for the family structure of the admixed cohort [30]. No further systematic covariates were fitted, because they were either constant or absent by design: all individuals belonged to a single contemporaneous cohort, were measured at the same age (18 mpf), and were reared communally in one pond under identical conditions, so that age and rearing-batch/environment effects did not vary; among-population ancestry was captured by the PCs, and residual genetic relatedness was modeled by the kinship matrix K. Sex was not fitted as a covariate. At 18 mpf, *C. alburnus* is still well before sexual maturity (males and females attain sexual maturity at approximately 2 and 3 years, respectively [36]), so that the gonads were too immature to permit reliable sex identification. At this pre-maturation stage, sexual size dimorphism is, in any case, negligible—body weight and length do not differ significantly between the sexes in *C. alburnus* [36]. Together with random mating, which makes sex largely independent of autosomal genotype, the omission of sex therefore reduces statistical power rather than biasing the associations.

The genome-wide significance threshold was determined using Bonferroni correction [37], calculated as *p* = 0.05/N, where N represents the total number of filtered SNPs. The suggestive association threshold was set at −log_10_(*P*) = 5. Manhattan plots and Q–Q plots were generated using CMplot (v4.5.1) [38]. To quantify any residual test-statistic inflation arising from population stratification and cryptic relatedness, the genomic inflation factor (λ_GC_) was calculated for each trait as the ratio of the median observed χ^2^ statistic to the median expected under the null hypothesis (0.4549, the median of a χ^2^_1_ distribution) [39]. A λ_GC_ value close to 1 indicates that the linear mixed model—incorporating the genome-wide kinship matrix and the leading principal components—adequately controlled for confounding, whereas mild departures above 1 are expected for highly polygenic traits even in the absence of stratification [40]. The phenotypic variance explained (PVE) by each significant SNP was calculated according to the following formula [41]:

(2) $$PVE=\frac{2\beta^{2}\cdot MAF\cdot \left(1-MAF\right)}{2\beta^{2}\cdot MAF\cdot \left(1-MAF\right)+se({\beta )}^{2}\cdot 2N\cdot MAF\cdot \left(1-MAF\right)}$$

where β is the effect size; MAF is the minor allele frequency; se is the standard error of the effect size; and N is the effective sample size. Based on PVE values, SNPs were classified into three categories: large-effect loci (PVE > 0.10), moderate-effect loci (0.01 ≤ PVE ≤ 0.10), and small-effect loci (PVE < 0.01) [42].

In addition to Bonferroni correction, the Benjamini–Hochberg (BH) method [43] was applied to control the false discovery rate (FDR) for BW and BL, the two traits for which genome-wide significant SNPs were identified. FDR-adjusted q-values were calculated using the p.adjust function in R (v4.4). SNPs with FDR < 0.05 were considered statistically significant after multiple testing correction. To further evaluate the robustness of the identified associations, a permutation test was performed for BW and BL following the approach of [44]. In each permutation, phenotypic values were randomly shuffled among individuals while genotypes and the kinship matrix were held constant, and GEMMA was re-run using the same LMM framework. This procedure was repeated 1000 times per trait, and the minimum *p*-value from each permutation was recorded to construct the empirical null distribution. The genome-wide empirical significance threshold was determined as the 5th percentile of this distribution (α = 0.05).

### 2.7. Candidate Gene Identification

Based on the topmouth culter reference genome annotation, candidate genes were extracted from the ±5 kb regions flanking each significant and suggestive SNP. This window was defined quantitatively from the genome-wide LD decay measured in our own population, in which *r*^2^ decayed to the background level (0.1) at approximately 2 kb (Section 3.3). Because the chosen half-window (5 kb) substantially exceeds this background-decay distance, it comfortably encompasses the local LD block around each lead SNP—and hence the variants most likely to tag the causal site—while still limiting the inclusion of unlinked genes. Genes falling within these windows are therefore reported as positional candidates that require subsequent functional validation rather than as proven causal genes. Functional annotation of candidate genes was performed using eggNOG-mapper (v2.1.13) [45] against the eggNOG database (v5.0). GO and KEGG pathway enrichment analyses were conducted using clusterProfiler (v4.18.4) [46] with the complete set of annotated protein-coding genes in the topmouth culter genome used as the background gene set. The Benjamini–Hochberg method was used for multiple testing correction, with *p* < 0.05 as the threshold for statistical significance.

## 3. Results

### 3.1. Descriptive Statistics and Distribution Analysis of Phenotypic Data

Descriptive statistics for the five growth-related traits of topmouth culter (*C. alburnus*) at 18 mpf are summarized in Tables S1 and S2. The mean values of BW and BWW were 315.08 ± 91.08 g and 294.73 ± 83.16 g, respectively, with coefficients of variation (CV) reaching 28.91% and 28.22%, indicating substantial phenotypic variation for these two traits, whereas the length and height traits (TL, BL, BH) were considerably less variable (CV 8.46–10.44%). The Shapiro–Wilk test indicated that TL (*p* = 0.995), BL (*p* = 0.994), and BH (*p* = 0.725) followed normal distributions, whereas BW and BWW showed slight departures from normality (*p* < 0.01) while retaining approximately bell-shaped distributions; the corresponding frequency histograms and Q–Q plots are provided in Figure S1. Overall, the five traits exhibited the broad, continuous variation characteristic of quantitative traits and were suitable for GWAS. All pairwise Pearson correlations among the five traits were positive and highly significant (*p* < 0.01) (Table 1): BW and BWW were almost perfectly correlated (*r* = 1.00, as expected because BWW was measured on the same individuals after evisceration), and BW correlated strongly with TL, BL, and BH (*r* = 0.93, 0.94, and 0.94, respectively), indicating that these traits may share a substantial common genetic basis.

### 3.2. Sequencing Data and SNP Identification

**Figure 1:**
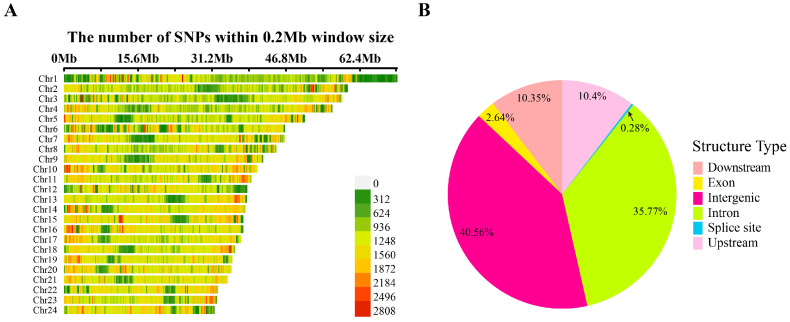
Genome-wide SNP distribution characteristics in topmouth culter. (**A**) SNP density distribution across 24 chromosomes (using 0.2 Mb windows). Different colors represent the corresponding number of mutations within a 0.2 Mb interval according to the legend. (**B**) Proportion of SNPs distributed across different genomic functional regions.

Whole-genome resequencing of 300 samples generated a total of 3948.55 Gb of clean reads, with an average sequencing output of 13.16 Gb per sample and average Q20 and Q30 rates of 97.73% and 92.81%, respectively (Table S3); the average sequencing depth was 11.44× (Table S4). The average mapping rate to the reference genome was 99.52%, with an average genome coverage (≥1×) of 85.79%, and an average genome coverage at ≥5× depth of 72.02%, ensuring the reliability of subsequent variant detection (Table S4). After stringent quality control, a total of 7,597,008 high-quality SNPs were obtained for GWAS analysis. The SNP density distribution across the genome, plotted using a 0.2 Mb window, showed a relatively uniform distribution of SNPs throughout the genome (Figure 1A), with chromosome 2 harboring the highest number of SNPs (407,247) and chromosome 23 the lowest (249,623). The average marker density across the genome was approximately 134.5 bp/SNP. The majority of SNPs were located in intergenic regions (40.56%) and intronic regions (35.77%), while 2.64% of SNPs were located in exon regions (Figure 1B). Regarding variant types, transitions accounted for 62.74% of total SNPs, comprising C > T (G > A) at 37.46% and T > C (A > G) at 25.28%; transversions accounted for 37.26%, including T > G (A > C) at 8.65%, C > G (G > C) at 5.14%, T > A (A > T) at 12.58%, and C > A (G > T) at 10.89%. The transition-to-transversion ratio (Ti/Tv) was 1.68, falling within the expected range and further validating the quality of the SNP data.

### 3.3. Population Structure Analysis

**Figure 2:**
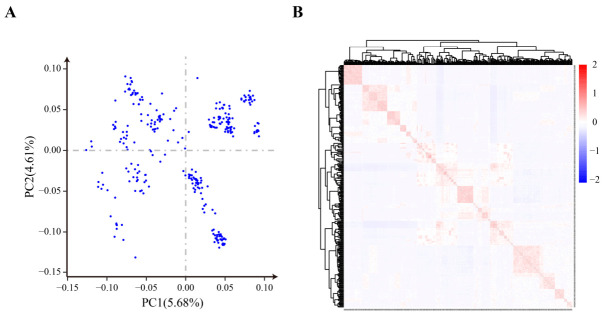
Genetic background of the experimental cohort of *C. alburnus*. (**A**) Principal component analysis (PCA) plot based on genome-wide SNPs; each point represents one individual, with the percentage of genetic variance explained shown on each axis; (**B**) Genomic kinship (relatedness) matrix among the 300 individuals; color intensity indicates the degree of pairwise relatedness.

**Figure 3:**
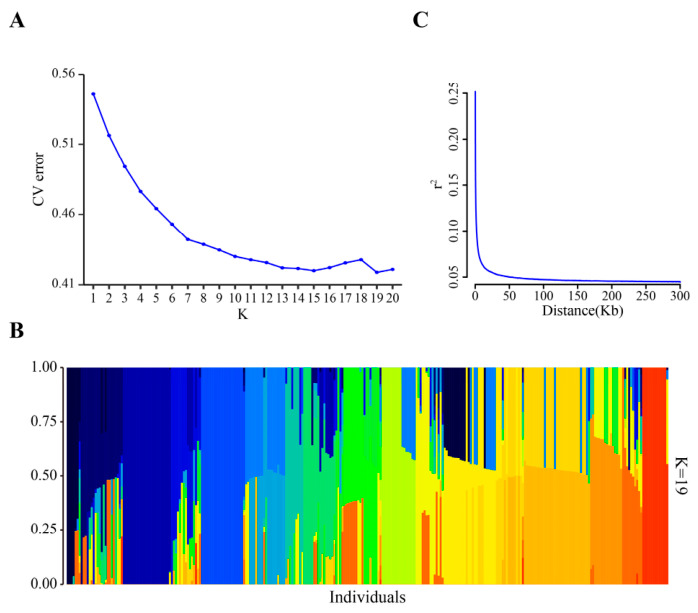
Population structure analysis of the topmouth culter experimental population. (**A**) Cross-validation (CV) error as a function of the number of assumed ancestral clusters K (1–20); the optimal K corresponds to the lowest CV error. (**B**) Population structure plot at K = 19. Each vertical bar represents one individual, and each color represents one of the 19 inferred ancestral genetic clusters; the length of each colored segment indicates the estimated membership (ancestry) proportion of that individual to the corresponding cluster. (**C**) Genome-wide linkage disequilibrium (LD) decay curve. The x-axis indicates physical distance (Kb) and the y-axis indicates the LD value (*r^2^*); the single curve shows the average rate at which LD decays with increasing physical distance.

To comprehensively evaluate the genetic background of the experimental population, PCA, kinship analysis, and population structure analysis were performed. PCA indicated only weak genetic substructure: the first two principal components together explained ~10.3% of the total genetic variance (PC1, 5.68%; PC2, 4.61%), and the individuals formed a single, loosely clustered cloud rather than discrete groups (Figure 2A). Consistent with this, the kinship matrix revealed structured relatedness within the cohort (Figure 2B). Population structure analysis showed that the CV error rate decreased continuously with increasing K values, from 0.5456 at K = 1 to 0.4421 at K = 7, after which the rate of decline slowed and reached a minimum of 0.4187 at K = 19 (Figure 3A). Because the experimental cohort was generated from only 131 wild-caught broodstock representing three populations, this large K most likely reflects fine-scale sibship and family structure within the offspring generation rather than 19 true ancestral populations (see §4.2). At the K = 19 clustering level, samples exhibited highly differentiated genetic backgrounds (Figure 3B). LD analysis revealed rapid LD decay in the experimental population. When *r*^2^ decayed to the background level (0.1), the physical distance was approximately 2 kb (Figure 3C). This rapid LD decay rate indicates that the population possesses high genetic diversity, which is favorable for precisely localizing association signals within narrow candidate regions.

### 3.4. Genome-Wide Association Study for Growth-Related Traits

**Figure 4:**
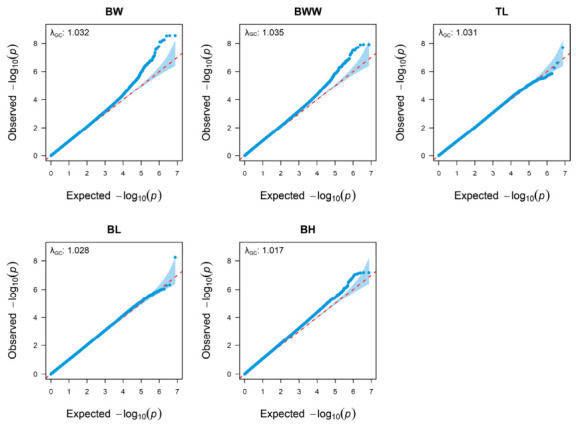
Q–Q plots of GWAS analysis for five growth-related traits in topmouth culter based on the linear mixed model (LMM). For each panel, the x-axis shows the expected –log_10_(p) values under the null hypothesis of no association, and the y-axis shows the observed –log_10_(p) values. The red dashed line denotes the expected distribution under the null hypothesis (the line of identity, y = x), and the blue shaded area indicates the corresponding 95% confidence interval under the null. Departure of the observed points above the dashed line and beyond the shaded band (upper-right tail) indicates true trait–marker associations. The genomic inflation factor (λ_GC_) is shown in the upper-left corner of each panel. BW: body weight; BWW: body weight without viscera; TL: total length; BL: body length; BH: body height.

**Figure 5:**
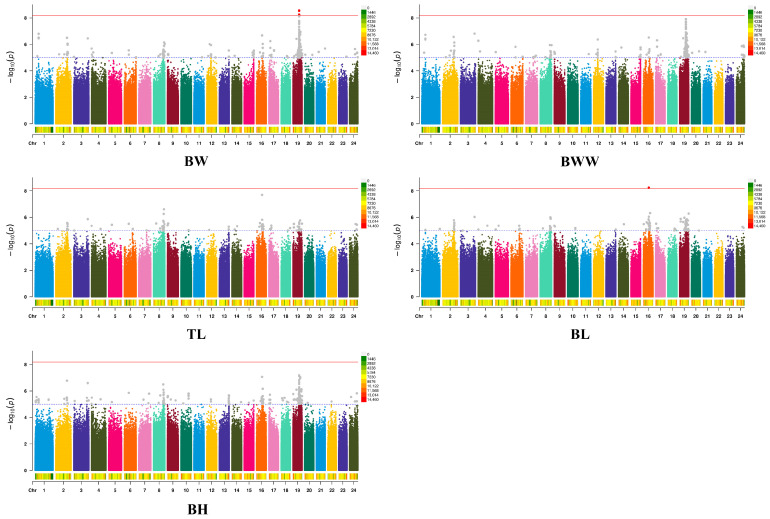
Manhattan plots of genome-wide association analysis for five growth-related traits in topmouth culter. The red solid line indicates the genome-wide significance threshold −log_10_(*P*) = 8.18, and the blue dashed line indicates the suggestive association threshold −log_10_(*P*) = 5. Numbers 1–24 represent chromosome numbers. BW: body weight; BWW: body weight without viscera; TL: total length; BL: body length; BH: body height.

GWAS was performed for the five growth-related traits of topmouth culter based on the LMM. Q–Q plots showed that when −log_10_(*P*) values were in the range of 0–3, the observed data points closely followed the expected diagonal; when −log_10_(*P*) exceeded 3, the observed points deviated upward from the diagonal (Figure 4), consistent with good model fit. Consistent with the Q–Q plots, the genomic inflation factors were close to unity for all five traits (λ_GC_ = BW, 1.032; BWW, 1.035; TL, 1.031; BL, 1.028; BH, 1.017; mean λ_GC_ = 1.029), with the corresponding values annotated on each panel of Figure 4. Based on Bonferroni correction, the genome-wide significance threshold was set at −log_10_(*P*) = 8.18 (P = 6.58 × 10^−9^). At this level, six SNPs were significantly associated with growth-related traits (Figure 5 and Table 2), all of which also passed Benjamini–Hochberg FDR < 0.05 (FDR = 0.004–0.040; Table S7). For BW, five significant SNPs were located on chromosome 19 with PVE > 11% and FDR = 0.004–0.005; the three lowest-FDR SNPs (Chr19:24066774, 24066776 and 24066777) lay immediately upstream of *aig1*. In total, 50 SNPs reached FDR < 0.05 for BW, of which 43 (86%) clustered within a ~19 kb interval on chromosome 19 (positions 24,057,508–24,076,475). Permutation testing (1000 replicates each for BW and BL) yielded empirical genome-wide significance thresholds of −log_10_*P* = 8.07 (BW) and 7.77 (BL) at *α* = 0.05, and all six Bonferroni-significant SNPs exceeded these empirical thresholds (Figure S2). An additional 291 suggestive SNPs were also detected for BW (Table S5). For BL, one genome-wide significant SNP (Chr16:21450218) was identified, with a PVE value of 11.05% and an FDR value of 0.040. No SNPs reached genome-wide significance for BWW, TL, and BH, although multiple associated loci were detected at the suggestive level. At the suggestive association level (6.58 × 10^−9^ < *p* < 10^−5^), a total of 946 associated loci were identified across the five traits (Figure 5 and Table S5), involving 473 independent loci after deduplication.

### 3.5. Candidate Gene Identification and Functional Analysis

**Figure 6:**
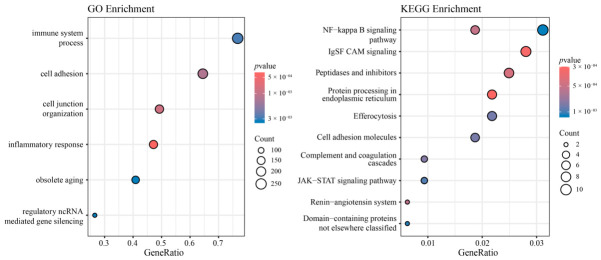
GO (**left panel**) and KEGG (**right panel**) enrichment analysis results for candidate genes in topmouth culter. *p*-value < 0.05.

Within 5 kb flanking regions of the 6 significant and 473 suggestive SNPs, a total of 350 candidate genes were detected (Table S6). Of these, 321 protein-coding genes were included in GO term and KEGG pathway analyses. GO enrichment analysis revealed enrichment of multiple growth-related functional categories, including immune system process, cell adhesion, and cell junction organization (Figure 6). KEGG enrichment analysis indicated that significantly enriched pathways included the NF-κB signaling pathway, IgSF CAM signaling, peptidases and inhibitors, protein processing in the endoplasmic reticulum, efferocytosis, and cell adhesion molecules (Figure 6). Based on significant SNP loci, four candidate genes—*aig1*, *cacna1b*, *pgm5*, and *bcr*—were identified as potentially associated with growth-related traits (Table 3).

## 4. Discussion

### 4.1. Phenotypic Variation and Breeding Potential

The substantially higher coefficients of variation (CVs) observed for BW and BWW (≥28%) compared with length and height traits (≤11%) within a single cohort reared under identical conditions indicate that genetic factors are the primary drivers of individual growth differences, implying considerable breeding potential. The strong positive correlations among all five traits (*r* > 0.90, *p* < 0.01) are consistent with the widely observed genetic correlations among growth-related traits in fish. These strong positive correlations suggest that selection for any single growth trait will generate correlated genetic gains in other traits, thereby simplifying breeding program design.

### 4.2. Population Structure and Statistical Model Validation

Accurate accounting for the genetic structure of the mapping population is a prerequisite for reliable GWAS, because uncontrolled relatedness and stratification can substantially inflate false-positive rates [30]. By design, the experimental fish were produced by communal natural (mass) spawning of broodstock intercrossed across the three source populations; the offspring therefore constitute a single admixed generation, in which geographic or ancestral subdivision is not expected. In the present cohort, the multidimensional analyses indicated only weak substructure by PCA—the first two principal components together explained ~10.3% of the genetic variance—and the dominant signal was fine-scale family (sibship) structure arising from the unequal contribution of broodstock under communal natural spawning, rather than geographic or ancestral population stratification (Section 3.3) [22,23]. To account for this relatedness, a linear mixed model was fitted with the first ten principal components as fixed-effect covariates [30] and the genomic kinship matrix as a random effect. The Q–Q plots indicated that this model adequately controlled the structure: in the low −log_10_(*P*) range, the observed statistics closely followed the null expectation, with departures confined to the extreme tail, where true association signals are expected. This visual assessment was corroborated quantitatively by genomic inflation factors close to unity for all five traits (λ_GC_ = 1.017–1.035; Section 3.4, Figure 4), confirming that test-statistic inflation due to stratification and cryptic relatedness was negligible. Together, these results indicate that the family structure and residual relatedness of the cohort were effectively corrected.

### 4.3. Identification of Growth-Related SNP Loci and Cross-Species Comparison

This study represents the first population-level GWAS conducted for topmouth culter. The six genome-wide significant SNPs were predominantly clustered on chromosomes 16 and 19, with PVE values ≥ 11.00%, suggesting the presence of important genomic regions harboring growth-regulatory elements on these two chromosomes.

The comparison of our results with those reported in other species reveals considerable variation in the genetic architecture of growth-related traits. Per-locus PVE values in topmouth culter are comparable to those reported in giant grouper (7.09–18.42%) [7] and brown-marbled grouper (*Epinephelus fuscoguttatus*, 10.24–15.46%) [47], but substantially higher than in rainbow trout (*Oncorhynchus mykiss*, 0.1–0.8%) [48], yellowtail kingfish (2.6–4.9%) [49], and Atlantic salmon (1.5–3.0%) [8], suggesting an oligogenic rather than highly polygenic architecture for growth in topmouth culter. In the closely related grass carp, Hao et al. [4] reported relatively smaller per-locus effects using a 21K SNP array, whereas Zhang et al. [5] identified loci of comparable magnitude through whole-genome resequencing. Regarding genomic distribution, the concentration of significant SNPs on only two chromosomes contrasts with the wider chromosomal spread observed in brown-marbled grouper (12 chromosomes) [50], giant grouper (14 chromosomes) [7], and grass carp [5]. Nevertheless, the 473 suggestive SNPs were distributed across all chromosomes, confirming that growth in topmouth culter is ultimately governed by multiple loci, consistent with the general principles of complex quantitative traits.

The robustness of these associations was further supported by three complementary criteria: Bonferroni correction, Benjamini–Hochberg FDR control, and permutation-based empirical thresholds (Figure S2). The permutation-derived thresholds were slightly more permissive than the Bonferroni threshold, reflecting the conservativeness of Bonferroni correction when neighboring SNPs are in linkage disequilibrium; nonetheless, all six significant SNPs exceeded all three thresholds (see Section 3.4 and Table S7), providing convergent evidence for the loci on chromosomes 16 and 19. LD analysis showed relatively rapid LD decay, with *r*^2^ reaching background levels at approximately 2 kb, which preserved detection power while improving the mapping resolution of candidate regions. Finally, the sample size of 239 individuals is moderate for aquaculture GWAS (typically 200–1000+ individuals) and, combined with 11.4× sequencing depth and 7.6 million high-quality SNPs, provided sufficient statistical power to detect moderate-to-large effect loci.

### 4.4. Candidate Genes Associated with Growth-Related Traits

A total of four candidate genes were identified within 5 kb flanking regions of significant SNPs. Based on their biological functions and SNP characteristics, three key candidate genes—*aig1*, *pgm5*, and *bcr*—are discussed in detail below.

*aig1* (androgen-induced gene 1) is the most noteworthy candidate gene in this study, showing strong associations with BW through five significant SNPs. AIG1 and its paralog ADTRP belong to a class of atypical transmembrane threonine hydrolases that utilize a conserved threonine-histidine catalytic dyad to specifically hydrolyze fatty acid esters of hydroxy fatty acids (FAHFAs) [51]. FAHFAs are a class of endogenous bioactive lipids with anti-inflammatory and insulin-sensitizing properties [52]. In mice, dual knockout of AIG1 and ADTRP led to significantly elevated FAHFA levels in brown adipose tissue, white adipose tissue, kidney, and liver [53], indicating that AIG1 plays an important role in lipid signaling metabolism and energy homeostasis. In fish, the cyprinid-specific fourth whole-genome duplication event (4R WGD) has endowed cyprinid fish with five AIG family gene copies [54], and gene copy expansion may confer more refined lipid metabolism regulation in cyprinid fish. In zebrafish, ADTRP has been demonstrated to regulate hematopoietic development through the TFPI-dependent signaling axis [55] and to participate in vascular development through the Wnt/*β*-catenin pathway [56]. Although direct functional studies of AIG1 in fish have not yet been reported, the above evidence supports the rationale for *aig1* as a candidate gene for growth-related traits in topmouth culter from multiple perspectives, including lipid metabolism regulation and cyprinid genome evolution.

*pgm5* is another important candidate gene. Despite its name suggesting an association with carbohydrate metabolism, biochemical studies have confirmed that PGM5 lacks phosphoglucomutase catalytic activity [57]. The actual function of PGM5 is as the cytoskeletal structural protein aciculin, localized at focal adhesions, stress fiber termini, cardiac intercalated disks, and skeletal muscle myotendinous junctions. Molt et al. [58] demonstrated that PGM5 is essential for myofibril assembly, remodeling, and maintenance, participating in sarcomere organization through interactions with filamin C and Xin proteins. PGM5 also directly binds dystrophin and utrophin [59], positioning it within core protein complexes for muscle development and integrity maintenance. In fish, Gustafsson et al. [57] reported that a missense mutation in the PGM5 gene showed significant allele frequency differences between Atlantic herring and Baltic herring populations adapted to different salinities. Given that muscle tissue constitutes the predominant proportion of body weight in fish, the structural function of PGM5 in myofibril assembly and maintenance provides a clear biological rationale for its association with body weight traits in topmouth culter.

The BCR protein contains three major functional domains: an N-terminal serine/threonine kinase domain, a central Dbl homology (DH) domain (functioning as a guanine nucleotide exchange factor for RhoA/Rac1/Cdc42), and a C-terminal GTPase-activating protein (GAP) domain (negatively regulating Rac1 and Cdc42) [60]. *bcr* knockout mice exhibited a three-fold enhancement of Rac2 membrane translocation [61], while the double knockout of BCR and ABR resulted in sustained Rac activation and significantly enhanced cell motility [62], indicating that BCR primarily functions as a negative regulator of Rac1 activity in vivo. Rac1 is essential for the G1-S phase transition of the cell cycle, directly influencing cell proliferation rates. Given the high conservation of Rho GTPase signaling pathways in vertebrates, the hypothesis that BCR participates in growth regulation in topmouth culter through modulating Rac1/Cdc42-mediated cell proliferation and differentiation pathways has a sound biological basis and warrants validation through subsequent functional experiments.

Notably, earlier candidate-gene studies in this species implicated classical growth regulators such as growth hormone receptor (*ghr*) and myostatin (*mstn*) [17,18], whereas our hypothesis-free genome-wide scan highlighted a distinct set of genes (*aig1*, *cacna1b*, *pgm5*, and *bcr*), illustrating the complementarity of candidate-gene and genome-wide association approaches.

### 4.5. Limitations

Several limitations temper the interpretation of these results. First, although the sample size of 239 individuals is moderate for aquaculture GWAS and, combined with stringent multiple-testing control (Bonferroni, Benjamini–Hochberg FDR < 0.05, and 1000-permutation thresholds), is adequate for detecting loci of moderate-to-large effect, it provides limited precision for estimating their effect sizes. The per-locus PVE (>11% for several SNPs) is therefore likely overestimated and should be regarded as an upper bound rather than an unbiased estimate—a phenomenon known as the Beavis effect [63,64]. Accordingly, the loci reported here are best interpreted as candidate loci of putatively moderate-to-large effect rather than as variants of precisely quantified effect size. Second, the associations were identified in a single synthetic, admixed cohort and have not been replicated in an independent population. Although the family structure and residual relatedness of this cohort were explicitly modeled through the leading principal components and a genome-wide kinship matrix, replication in independent genetic backgrounds remains essential before these loci can be confidently deployed in marker-assisted selection. Third, sex was not recorded and was therefore not fitted as a covariate. *C. alburnus* can exhibit female-biased growth in mature stocks [65], so sex is in principle relevant to adult body size. However, the experimental cohort was sampled at 18 mpf, well before sexual maturity in this species (~2 years in males and ~3 years in females [36]); when dimorphism is minimal and population-level length/weight differences are non-significant [36], and random mating makes sex largely independent of autosomal genotype, so its omission mainly reduces power rather than biasing associations. Future work will prioritize replication in independent populations, larger cohorts for effect-size estimation, recording of sex, and functional validation of candidate genes.

## 5. Conclusions

This study presents the first population-level GWAS of growth-related traits in topmouth culter (*Culter alburnus*), based on whole-genome resequencing of 300 individuals at an average depth of 11.4×. From 7,597,008 high-quality SNPs, 6 genome-wide significant and 473 suggestive SNPs were identified, with significant loci predominantly clustered on chromosomes 16 and 19; several SNPs each explained > 11% of the phenotypic variance, but because such estimates are prone to upward bias at this sample size (the Beavis effect), these are interpreted as candidate loci of putatively moderate-to-large effect that warrant validation in independent populations. Four candidate genes—*aig1*, *cacna1b*, *pgm5*, and *bcr*—were prioritized based on functional relevance to lipid metabolism, muscle structure, and cell proliferation. The robustness of these associations was supported by three independent statistical criteria (Bonferroni correction, Benjamini–Hochberg FDR, and 1000-permutation testing). Future work will focus on functional validation of the identified candidate genes—particularly *aig1*—through gene expression analyses (e.g., qRT-PCR and RNA-seq) and CRISPR/Cas9-mediated functional experiments, as well as verification of the SNP markers in independent populations across different developmental stages and rearing conditions. Given that relatively mature breeding systems (including gynogenetic all-female lines and distant hybridization lines) have been established for topmouth culter, integration of these molecular markers with existing breeding technologies is expected to accelerate the development of superior varieties and advance molecular breeding programs for this economically important species.

## Figures and Tables

**Table 1:** Pearson correlation coefficients among the five growth-related traits of *C. alburnus* (*n* = 239). All correlations are significant at *p* < 0.01.

Trait	BW	BWW	TL	BL	BH
BW	1.00				
BWW	1.00	1.00			
TL	0.93	0.93	1.00		
BL	0.94	0.99	0.99	1.00	
BH	0.94	0.94	0.92	0.93	1.00

**Table 2:** Summary of six SNPs significantly associated with growth-related traits of *Culter alburnus*.

Chr	Position	Beta	−log_10_(*P*)	PVE%	FDR	Allele	Traits
16	21450218	−1.7147	8.244	11.05	0.040	T/C	BL
19	24058522	64.0034	8.218	11.00	0.005	G/C	BW
19	24066756	65.3279	8.254	11.27	0.005	A/T	BW
19	24066774	66.5747	8.540	11.66	0.004	A/T	BW
19	24066776	66.4125	8.558	11.63	0.004	C/T	BW
19	24066777	66.4125	8.558	11.63	0.004	A/G	BW

Note: Chr, chromosome; Position, SNP position on the chromosome; Beta, effect size; −log_10_(*P*), association significance; PVE%, percentage of phenotypic variance explained; FDR, Benjamini–Hochberg false discovery rate; Allele, reference/alternate alleles; Traits, associated traits.

**Table 3:** Candidate genes related to growth-related traits.

SNP_ID	Gene_Name	Location	Gene_Description
Chr16:21450218	*pgm5*	Downstream	phosphoglucomutase 5
Chr16:21450218	*bcr*	Intron	BCR activator of RhoGEF and GTPase
Chr19:24058522	*cacna1b*	Upstream	calcium voltage-gated channel subunit alpha1 B
Chr19:24058522, 24066756, 24066774, 24066776, 24066777	*aig1*	Upstream	fatty acid esters of hydroxy fatty Acids hydrolase AIG1, androgen-induced gene 1 protein, FAHFA hydrolase AIG1

Note: SNP_ID was shown as ‘chromosome: position’.

## Data Availability

The whole-genome resequencing raw data generated in this study have been deposited in the Genome Sequence Archive (GSA) at the China National Center for Bioinformation (CNCB) under BioProject accession number PRJCA059576; to be provided during review. All accession numbers will be made publicly available prior to publication.

## Supplementary Materials

The following supporting information can be downloaded at: https://www.mdpi.com/article/10.3390/ani16131969/s1, Figure S1: Phenotypic data distribution of the five growth-related traits at 18 mpf (frequency histograms and Q–Q plots); Figure S2: Empirical null distributions of minimum *p*-values from 1000 permutations for body weight (A) and body length (B) in *Culter alburnus*; Table S1: Summary of phenotypic information of 239 samples; Table S2: Trait statistics summary; Table S3: Summary of whole-genome resequencing quality for each sample; Table S4: Summary of whole-genome resequencing mapping efficiency and genome coverage for each sample; Table S5: Summary of 946 SNP suggestive associated with growth-related traits of *Culter alburnus*; Table S6: Summary of SNPs and candidate genes associated with growth-related traits of *Culter alburnus*; Table S7: SNPs significantly associated with growth-related traits after Benjamini–Hochberg FDR correction (FDR < 0.05) in *Culter alburnus*.

## Abbreviations

The following abbreviations are used in this manuscript:

BW	Body weight
BWW	Body weight without viscera
TL	Total length
BL	Body length
BH	Body height
GWAS	Genome-wide association study
SNP	Single-nucleotide polymorphism
LD	Linkage disequilibrium
LMM	Linear mixed model
MAF	Minor allele frequency
PCA	Principal component analysis
PVE	Phenotypic variance explained
FDR	False discovery rate
CV	Coefficient of variation
QTL	Quantitative trait locus
MAS	Marker-assisted selection
GS	Genomic selection
PIT	Passive integrated transponder
mpf	Months post-fertilization
