# Supplementary material for: Whole-Genome Resequencing-Based GWAS Reveals Major-Effect Loci and Candidate Genes for Growth Traits in Topmouth Culter (Culter alburnus)
Source: Animals (Basel). 2026 Jun 25;16(13):1969. doi: 10.3390/ani16131969 (PMC13360439; doi:10.3390/ani16131969)
Supplement: Supplementary file 1 [file animals-16-01969-s001.zip › Supplemental_Figures.pdf]

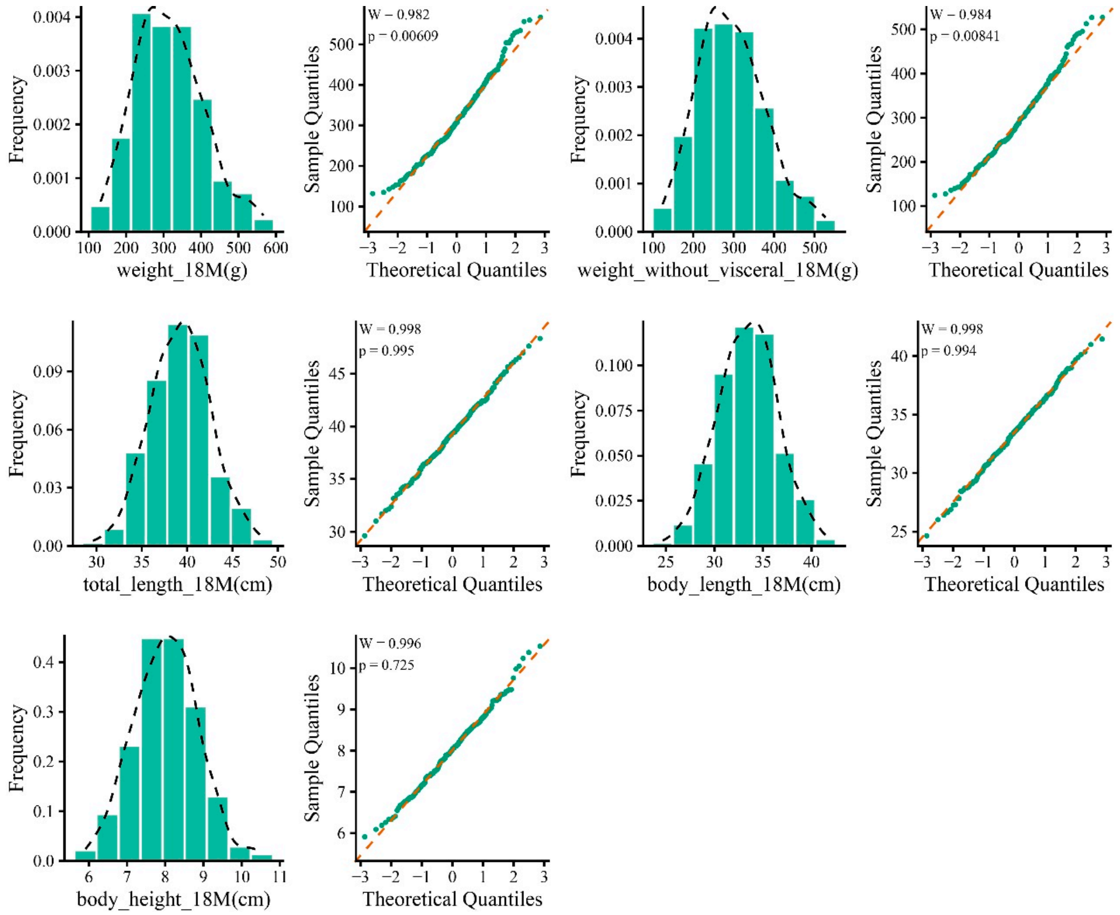

Figure S1. Phenotypic data distribution of five growth traits in topmouth culter at 18 mpf. Left panels show frequency distribution histograms (overlaid with normal distribution curves), with the X-axis representing trait measurement values and the Y-axis representing frequency; right panels show Q-Q plots. BW: body weight; BWW: body weight without viscera; TL: total length; BL: body length; BH: body height.

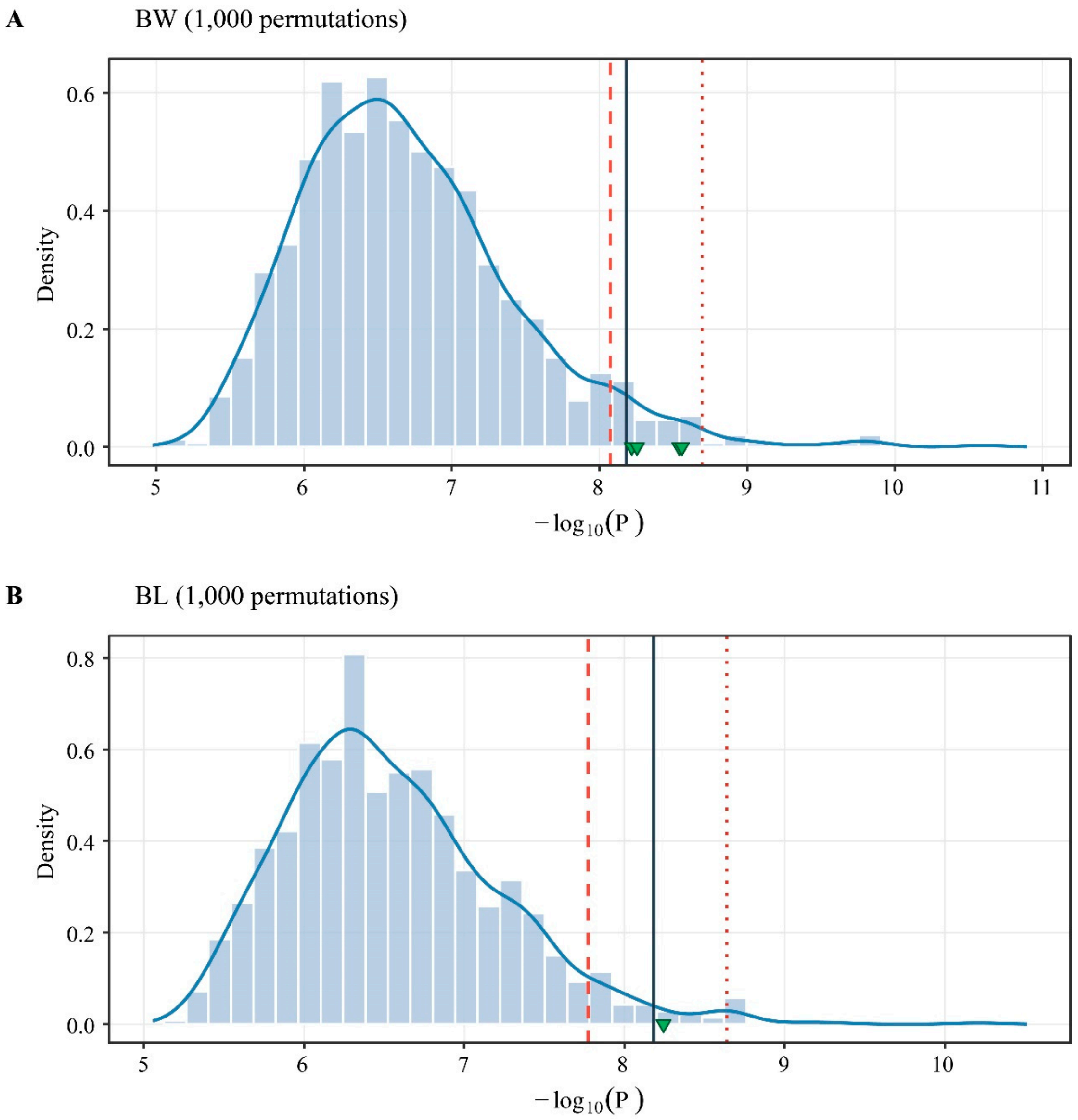

Figure S2. Empirical null distributions of minimum *P*-values from 1,000 permutations for body weight (A) and body length (B) in *Culter alburnus*. Histograms with density overlays show the distribution of minimum $-\log_{10}P$ values across permutations. The solid vertical line marks the Bonferroni genome-wide significance threshold ($-\log_{10}P = 8.182$); the dashed and dotted lines mark the empirical thresholds at α = 0.05 and α = 0.01, corresponding to the 5th and 1st percentiles of the permutation distribution, respectively. Green triangles indicate the observed $-\log_{10}P$ values of the genome-wide significant SNPs from the original GWAS. All six SNPs exceeded the empirical α = 0.05 threshold, supporting the robustness of the associations.
